# Participation in a single-blinded pediatric therapeutic strategy study for juvenile idiopathic arthritis: are parents and patient-participants in equipoise?

**DOI:** 10.1186/s12910-018-0336-8

**Published:** 2018-12-20

**Authors:** Petra C. E. Hissink Muller, Bahar Yildiz, Cornelia F. Allaart, Danielle M. C. Brinkman, Marion van Rossum, Lisette W. A. van Suijlekom-Smit, J. Merlijn van den Berg, Rebecca ten Cate, Martine C. de Vries

**Affiliations:** 1 Department of Pediatric Rheumatology, Leiden University Medical Center, PO Box 9600, 2300 RC Leiden, the Netherlands; 2 Department of Rheumatology, Leiden University Medical Center, Leiden, the Netherlands; 3 Department of Pediatrics, Alrijne Hospital, Leiderdorp, the Netherlands; 4 Department of Pediatric Rheumatology, Reade Amsterdam Rheumatology Center, Amsterdam, the Netherlands; 5 Department of Pediatric Rheumatology, Erasmus MC Sophia Children’s Hospital, Rotterdam, the Netherlands; 6 Department of Pediatric Hematology Immunology Infectious Diseases and Rheumatology, Emma Children’s Hospital/Academic Medical Center, Amsterdam, the Netherlands; 7 Department of Medical Ethics and Health Law, Leiden University Medical Center, Leiden, the Netherlands

**Keywords:** Equipoise, Clinical trial, Juvenile idiopathic arthritis, Therapeutic strategy study, Biologicals, Treatment-to-target, Tight control, Individual, Patient-group, Randomization, Pediatric rheumatology, Informed consent

## Abstract

**Background:**

Genuine uncertainty on superiority of one intervention over the other is called equipoise. Physician-investigators in randomized controlled trials (RCT) need equipoise at least in studies with more than minimal risks. Ideally, this equipoise is also present in patient-participants.

In pediatrics, data on equipoise are lacking. We hypothesize that 1) lack of equipoise at enrolment among parents may reduce recruitment; 2) lack of equipoise during participation may reduce retention in patients assigned to a less favoured treatment-strategy.

**Methods:**

We compared preferences of parents/patients at enrolment, documented by a questionnaire (phase 1), with preferences developed during follow-up by an interview-study (phase 2) to investigate equipoise of child-participants and parents in the BeSt-for-Kids-study (NTR 1574). This trial in new-onset Juvenile Idiopathic Arthritis-patients consists of three strategies. One strategy comprises initial treatment with a biological disease-modifying-antirheumatic-drug (DMARD), currently not standard-of-care. Semi-structured interviews were conducted with 23 parents and 7 patients, median 11 months after enrolment.

**Results:**

Initially most parents and children were not in equipoise. Parents/patients who refused participation, regularly declined due to specific preferences. Many participating families preferred the biological-first-strategy. They participated to have a chance for this initial treatment, and would even consider stopping trial-participation when not randomized for it. Their conviction of superiority of the biological-first strategy was based on knowledge from internet and close relations. According to four parents, the physician-investigator preferred the biological-first-strategy, but the majority (*n* = 19) stated that she had no preferred strategy. In phase 2, preferences tended to change to the treatment actually received.

**Conclusions:**

Lack of equipoise during enrolment did not reduce study recruitment, mainly due to the fact that preferred treatment was only available within the study. Still, when developing a trial it is important to evaluate whether the physicians’ research question is in line with preferences of the patient-group. By exploring so-called ‘informed patient-group’-equipoise, successful recruitment may be enhanced and bias avoided.

In our study, lack of equipoise during trial-participation did not reduce retention in those assigned to a less favoured option. We observed a change for preference towards treatment actually received, possibly explained by comparable outcomes in all three arms.

**Electronic supplementary material:**

The online version of this article (10.1186/s12910-018-0336-8) contains supplementary material, which is available to authorized users.

## Background

It is an ethical requirement that physician-investigators provide research-participants the best treatment available in randomized controlled trials (RCT) [1–3]. In a clinical trial comparing different treatment strategies there should be uncertainty regarding preferred treatment option considering therapeutic efficacy and safety [1–4]. This is called equipoise. ‘Individual equipoise’ implies that the *individual* physician-investigator must possess this genuine uncertainty [1]. Affected by preliminary results during a trial, a physician-investigator could develop a preference which might lead to a perceived conflict whether the best treatment known is actually provided. Therefore, Freedman described ‘clinical equipoise’ as genuine uncertainty in the *medical expert community* instead of genuine uncertainty in the individual physician-investigator [1]. Clinical equipoise allows the physician-investigator to collect evidence to convince the expert community of either superiority. Critics on clinical equipoise argue that this concept needs to be transformed to adapt to forward modern health care: if the potential social value of a study is relevant and participants are not exposed to excessive net risks, clinical equipoise can be amended [5, 6]. Kimmelmann questions whether equipoise should be rethought as a prima facie principle rather than an absolute one [7] and we agree on that. Miller et al. even question the necessity of equipoise [8].

Literature suggests that not only the medical expert community should be in equipoise but also patient-participants (and their parents, if children are concerned) [8, 9].

In pediatrics, experience with clinical equipoise is limited [10, 11]. In pediatric oncology, parental and physician equipoise has been scarcely studied [12, 13]. Difficult protocols, strong emotions and the parents’ dependency on their child’s physician are reasons for often lacking parental equipoise. In pediatric rheumatology, treatment preferences among physicians have been studied by Hugle et al. [14] revealing that availability and funding influenced physicians’ choices. A discrete choice experiment explored parents preferences in juvenile idiopathic arthritis in daily clinical care [15]. Parents have strong preferences for treatments that reduce pain and improve daily functioning, regardless of side effects. With increasing disease duration, parents preferences focused on therapeutic effectiveness. Little is known about patient-participant-equipoise or parent-equipoise [9, 12, 16] in pediatric clinical research on chronic diseases. More insight in this equipoise is particularly important when considering the inherent vulnerability of children in research [17].

## Methods

### Aims

In this study we aimed to evaluate (1) the preferences of Juvenile Idiopathic Arthritis (JIA) patients aged 12 years and older, and their parents for a certain treatment strategy in the setting of a randomized clinical trial, and (2) the influence of the informed consent procedure on these preferences.

### Context BeSt for kids study

The trial concerned *the BeSt for Kids study* (Dutch Trial Register NTR 1574), a multicenter, randomized, single-blinded two year follow-up clinical trial comparing time- to-inactive disease and time-to-flare in selected categories of newly diagnosed JIA patient-participants. In this study patient-participants (age between 2 and 16 years) with a maximum of 18 months of complaints and 1) oligoarticular JIA 2) Rheumatoid Factor (RF) negative polyarticular JIA and 3) Juvenile Psoriatic Arthritis, (6) with active disease requiring treatment with a disease modifying antirheumatic drug (DMARD) according to the treating pediatric rheumatologist were randomized in three treatment strategies. These strategies are.

1. initial monotherapy with sulfasalazine or methotrexate,
2. initial combination therapy with methotrexate and prednisone bridging,
3. initial combination therapy with methotrexate and etanercept.

In the study protocol subsequent steps to reach inactive disease are dictated in case of insufficient response in all three arms. An additional file contains the summary of the protocol initial treatments and subsequent treatment steps [see Additional file 1].

Data on efficacy of the different individual DMARD is available in literature [18–20]. No data existed before and during inclusion on the superiority of either of those strategies.

The treatment was single-blinded: the periodic assessment of disease activity was performed by a physiotherapist, unaware of the allocated treatment, but patient and physician were not blinded.

The informed consent procedure for inclusion in the BeSt for Kids study consisted of at least one visit to the outpatient clinic, with an oral explanation by the attending physician and the research nurse and complementary written information. In addition to the patient- subjects information form (PIF), which is available as supplementary file [see Additional file 2], all newly diagnosed patients with juvenile idiopathic arthritis are referred to http://www.printo.it for general information on juvenile idiopathic arthritis. Besides that, patients and parents had several days to week(s) between receiving written PIF and actual enrolment. The BeSt for Kids was approved by the Institutional Review Board at Leiden University Medical Center and written informed consent was obtained from all participants before enrolment.

### Design equipoise study

#### Phase 1 (questionnaire)

When parents and children (aged 12 years and older) consented to participate in the trial, they were randomized. Subsequently the physician completed the Case Report Form which included a questionnaire asking parents and patients for their preferred strategy before actual allocation to the strategy. This questionnaire has been added as supplementary file [see Additional file 3]. To diminish bias by only asking participating parents and patients we additionally collected the reasons for not participating in the study.

#### Phase 2 (interview study)

We conducted an interview study with parents and patient-participants aged 12 years and older participating in the BeSt for Kids study which was designed after of the onset of the study. Parents and patient-participants were informed of the interview by a letter asking them to participate in the interview study. All patients enrolled in the study at that time point (*n* = 29) were contacted by telephone for participation, which lead to an appointment for an interview with a short questionnaire. This questionnaire is added as supplementary file [see Additional file 4]. To facilitate families, the actual interview was held in the hospital or at home by choice. One-to-one, semi-structured interviews were conducted with the parents and patient-participants.

We choose the age of 12 years for patient interviews since in the Netherlands by law children from the age of 12 years old are actively involved in their healthcare related decisions in consultation with their parent(s) or guardian(s).

This study and the consent procedure were approved by the IRB of the Leiden University Medical Center. Verbal consent was obtained from both parents and children, according (no capital needed) to the Dutch Law (Medical Research Involving Human Subjects Act (WMO) and Medical Treatment Agreement Act (WGBO) for studies with negligible risk and burden only verbal consent is necessary.

### Interview procedure and analysis

All parents and patient-participants were interviewed by researcher B.Y. Interview topics and questions were formulated after evaluation of the relevant literature. Topics were: 1 Evaluation of the Informed consent procedure, 2 Preference for treatment strategy, 3 Comments on preference, 4 Impression of physicians’ preference and 5 Main reasons for participation in BeSt for Kids study. Interviews contained closed-ended as well as open-ended questions. Using the latter, participants could elaborate on their answers on closed-ended questions. Interviews lasted between 20 and 45 min.

The interviews were recorded and transcribed verbatim. Data analysis of the interviews was based on the constant comparative method [21, 22]. One of the researchers encoded the full transcripts manually by identifying and labeling discrete units of texts which refer to one or more concepts relevant to the study. Through comparison across transcripts, open codes were developed into higher order themes to provide a framework for coding subsequent transcripts. P.H.M. and M.d.V. coded a random sample of the interviews to check for consistency and adequacy of the framework. When no new thematic content was found in the parent interviews, subject enrolment was stopped. This process, called thematic saturation, is a well-described qualitative method to avoid unnecessarily large and repetitive data sets [23, 24].

Finally, representative quotations from parents and physicians were chosen to demonstrate the themes identified.

## Results

### Phase 1 questionnaire: Preference at inclusion in the BeSt for kids study

During recruitment, we have received information on reasons for refusal in 15 out of 36 refusals (Table 1). In total 94 children were randomized in the BeSt for Kids study.

**Table 1:** Summary of patients/parents who refused to participate in the BeSt for Kids study with reasons for refusal

Number of patients who refused to participate	*n* = 36
Known reason for refusal of trial participation	*n* = 15 (42%)
Preference for arm 1	1
Preference for arm 3	1
Fear in general	1
Do not want to randomize at all	2
Do not want to receive prednisone or injections	2
Do not want to receive arm 3	2
Too busy to participate	2
Don’t feel like it	2
No reason mentioned when asked	2

All parents and children aged 12 and older were asked during enrolment in the BeSt for Kids study, before randomization, whether they hoped to be assigned to a particular treatment strategy. At the start 46% of parents of all enrolled patient-participants (*n* = 94) expressed to have no preference, 34% hoped for assignment to strategy 3 (initial etanercept with methotrexate) and 7% hoped against assignment to strategy 3. Primary aversion was highest for the second strategy (25%) due to a dislike of prednisone (data not shown). To compare, reasons for refusal to participate in the study were documented in 15/36(42%) and are summarised in Table 1. Multiple reasons were expressed, ranging from strong preference to dislike for a particular arm. Six out of 36 (17%) expressed explicit preferences or dislike of arm 1 or arm 3.

### Phase 2: Interview study

**Fig. 1:**
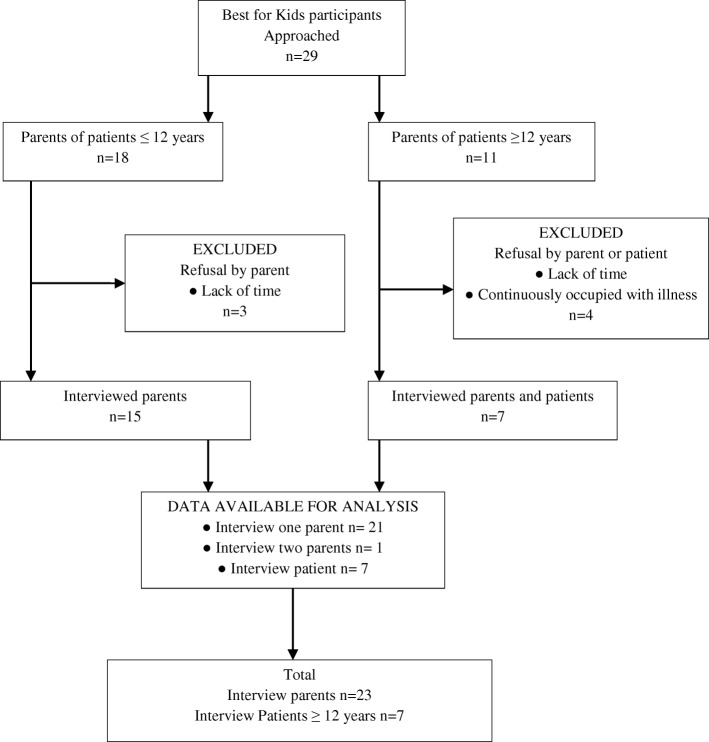
Recruitment of parents and patients

Figure 1 shows the recruitment of parents and patient-participants in our interview study. Twenty-nine patients were approached for the interview-study, finally 23 interviews were conducted with 22 mothers and 1 father. Parents had a mean age of 40.0 years (range 32–51 years) and patient-participants 14.3 years (range 12–17 years). All participants were Dutch speaking. Two parents were divorced, the rest was married. The education level was middle level high school (*n* = 1), high level high school (*n* = 1) intermediate vocational (n = 1), secondary vocational (*n* = 8) and advanced vocational/university (*n* = 12).

Between inclusion in the BeSt for Kids study and the interview (Phase 2) was a period of mean 11.8 (3.4–19.1) months.

Parent 14 and 15 are parents of the same patient. In parent number 1–16 only parents were interviewed. In parent number 17–23 both parent and patient were interviewed.

The concepts that were identified in the qualitative analysis resulted in a framework that comprises the following three themes which will be discussed separately:

I. Non-participation or withdrawal is not without consequences.
II. The conviction of superiority of the ‘experimental’ strategy.
III. Participation is in the best interest of the child.

### Theme 1: Non-participation or withdrawal is not without consequences

The majority of parents and children was well aware of the study design. Results indicated no differences between parental and patient-participants’ understanding concerning study name, duration, aim and number of treatment strategies. Although almost all participants (parents *n* = 23, patient-participants *n* = 7) knew that they were allowed to withdraw from the study at all times, seven parents and four patient-participants believed that stopping the trial would have consequences for the treatment in terms of less quality and quantity of the patient-participant care.

*(Parent 8) Sure it has consequences. This means he is not being looked after […].*

*(Parent 18) Now she gets such good care. That would be less; there would be less time and less checkups.*

Two parents and three patient-participants argued that it would have consequences because initial treatment with etanercept is not covered by insurance companies outside the trial.

*(Parent 19) Yes I think so. If you stop then you receive no further medication.*

*(Patient-participant 17) It could be that the insurance company requires you to pay for the drugs.*

*(Patient-participant 23) Because if I stop it has consequences for my treatment. Because I could never get this treatment by the insurance.*

### Theme 2: The conviction of superiority of the experimental strategy

All parents (*n* = 23) expressed that they had a preference for a particular treatment strategy. Fourteen parents (61%) preferred the third strategy (initial etanercept with methotrexate) whereas 3 (13%) preferred the first strategy (initial sulfasalazine or methotrexate), one parent preferred the second strategy (initial methotrexate and prednisone bridging), one parent preferred the first or second strategy and four (17%) preferred a non-prednisone strategy.

Five of seven patient-participants had a preferred strategy. Three of them preferred initial etanercept with methotrexate (third strategy) and two preferred a non-prednisone strategy. Two of the patient-participants had no preference.

Generally, as main explanation for their preference for the 3rd strategy (initial etanercept with methotrexate) parents and patient-participants stated that they believed it is the best treatment for JIA given the results of previous studies [25–28].

*(Patient-participant 23) I also wanted that drug (etanercept), even though we did not know the side effects. Still, if you hear that it works very well and that the arthritis completely disappears from your joints, and therefore it is the best drug, then it seems obvious to me that you would want that.*

*(Parent 3) At one moment I read on the internet an experience by a mother who said she finally got her teenage daughter back, this was not the crucial reason but at that moment it confirmed my gut feeling.*

*(Parent 18) Well we have been searching quite a lot on the internet and it just gives reasonably good results as far as they presently know.*

Parents and patient-participants indicated that these beliefs were mainly based on knowledge they had gained through the internet and from experiences in their environment.

For both parents and patient-participants reluctance to prednisone was due to well-known side-effects, mainly gaining weight.

*(Patient-participant 19) What I didn’t want was prednisone, actually. That’s because my mother had to use it for a long time and I have seen what it does, it has a lot of severe side effects.*

The majority of parents (*n* = 19) mentioned that the physician did not express a preferred treatment strategy. Four parents stated that the physician preferred etanercept-first strategy.

*(Parent 6) The physician had no preference. She indicated that she was very curious about what the outcomes of the study will be.*

*(Parent 9) Yes, that new medicine. That’s what she really said. She literally said that they would like to give it to us. But that simply couldn’t, because of the study and because insurance companies do not want to pay.*

*(Parent 14a) I thought that the physician absolutely had no preference at all.*

*(Parent 16) Because of course, I didn’t know it yet, we were confronted with a diagnosis that really was unexpected. [ … ] So I had the impression that they were very enthusiastic about this new drug, and that the study gave us the opportunity to get it earlier.*

One of the patient-participants thought that the physician preferred initial treatment with etanercept. One did not remember and five recalled that the physician did not have a preference.

### Theme 3 participation is in the best interest of the child

Although some families stated that they (also) participated in the BeSt for Kids study to help the good cause of research and therefore to support the next generations of JIA patients (*n* = 6 parents), many families expressed personal reasons to participate.

*(Parent 4) […] It was more the extra attention[….] However, also a little bit for the good cause, of course, that other patients could benefit from it as well in the long term.*

Seven parents joined the study because they assumed that in participating, their child would be more closely observed. Parents also participated for the best prospect of their child (*n* = 5), hoping that joining the study was the best they could do. Five parents stated that their reason for participation was to have the opportunity for initial treatment with etanercept (strategy 3). One mother even expressed that she would have withdrawn from the trial if her child had not randomized in their preferred strategy.

*(Parent 23), Had I drawn strategy 2, I would have immediately stopped. Then I would have chosen my own direction with my child, off course in consultation with the physician In that case I would not want to participate in the study, and then you should have to work with the available resources.*

*(Parent 5) [… .]Of course there are many benefits as a result of taking part …*

Patient-participants also joined the study because of a chance for initial treatment with etanercept (strategy 3)(*n* = 3).

*(Patient-participant 23) I believe that our main goal was that we could get the really good medication.*

Two patient-participants only mentioned their wish to recover (*n* = 2) without giving another reason for participation. Two patient-participants chose to participate for the good cause of research.

#### Changing preferences

When comparing the results from phase 1, before randomization, to the results during the interview (phase 2), half of the parents (11/23) showed a different preference in the interview compared to their opinion at enrolment, from which 6 out of 11 changed to preference of the actual enrolled treatment strategy, mainly increasing preference for arm 1 and arm 3. Eight out of 23 (36%) had a persistent preference for strategy 3. Two tables illustrate the changing preferences according to the treatment actually enrolled to [see Additional file 5: Tables S2 and S3].

## Discussion

The results of our interview study demonstrate that at enrolment (phase 1) many parents and children in the BeSt for Kids study are not in equipoise, because most of them hold the conviction that strategy 3, initial combination therapy with methotrexate and etanercept, is medically superior to the other strategies, as described in theme 2. In the majority of parents this is not caused by an assumed preference of the physician-investigator but by information on the various treatment possibilities obtained from other sources.

This is an evaluation of parents’ preferences for treatment strategies by a questionnaire at enrolment (phase 1) as well as by interviews several months into the different treatment strategies (phase 2). When comparing the preferences at the two time points we conclude that parents increasingly preferred initial combination therapy with etanercept and methotrexate and disliked taking prednisone.

Having a preferred strategy in general increased from 62% at enrolment to 100% of parents during the study period. This difference can be explained by the fact that the interview took place almost a year after study enrolment so that perception can be modified by experience. Initially preferences focused on fear of side effects of prednisolone and suspected superiority of the initial etanercept (arm 3). In phase 2 preferences shifted to mainly arm 1 and arm 3, often the actual strategy children received. Parents and children by then seemed to focus on effectiveness of the therapy received, as was described previously [15]. This result is also consistent with previous results in the BeSt-trial in rheumatoid arthritis patient-participants [29] where patients clearly preferred initial combination therapy with infliximab and disliked taking prednisone.

Parents have many motivations when deciding whether or not to let their child enter a randomized clinical trial. They will not easily agree on randomization because an ethics committee has approved the study [30, 31]. The primary responsibility of parents is to act in (what they think is) the best interest of the child, and the choice to enter a trial is based both on ‘objective‘probabilities of trial outcomes and on the value that parents and patient-participant place on those outcomes [32]. Also in our study, the main reason for parents to participate in the BeSt for Kids study was to support the best interest of their child (theme 3). For some parents, the trial represented the prospect of receiving a new, not routinely available treatment with a potentially important direct benefit to their child as was recognized previously as an important motivator for parents and patients to participate in studies [30, 33]. Etanercept is in the Netherlands not reimbursed as first treatment option. Therefore, as initial treatment, it was only available within the trial and parents may consent to their child’s entry because of the chance of receiving these assumed benefits [30, 34]. It may cause them firstly to anticipate remorse for not at least trying to obtain this new treatment through trial participation, and secondly to expect consequences when withdrawing during the study. This is understandable from their perspective as guardian of the interests of their child [12]. One could imagine a different outcome in cases where parents prefer a standard treatment which is routinely available outside of research. Additionally, a short course of Prednisolone is regularly applied in daily patient care in Juvenile idiopathic arthritis patients as bridging therapy, since methotrexate is a slow-acting DMARD.

Whatever trial strategy parents think is better for their child, their preference shows that the idea of clinical equipoise held by the expert medical community is not directly transferable to the parent setting as a proxy [35]. For parents and children the different strategies of a trial are often not in equipoise, because they hold the conviction that one strategy is medically superior [33, 34, 36]. Although in this study many parents, but not all, were prepared to enter a study (and continue participation) when they had preferences for therapy other than what they received, this lack of equipoise could be a problem for recruitment in RCT’s.

Recently it was suggested by Whybrow [35] to take an epidemiological approach to the concept of equipoise situating it as a measurable characteristic of a target patient-group. We argue that both types of patient equipoise (*individual* and *patient-group*) are relevant at different time points in the clinical research setting. Individual patient equipoise is relevant when actually contemplating trial participation between physician and patient/parent. Individual values need to be discussed and exchanged to explore possible trial participation. Informed patient-group equipoise is relevant, and we would say even necessary, in the phase of developing a trial to evaluate whether the research question the physician wants to answer is in line with the preferences of the patient group.

If scientists are aware of the consequences of strong patient preferences by evaluating the patient group equipoise they can anticipate to the possible lack of inclusions in a trial. One example with a deviating clinical expert equipoise and patient group equipoise is the CONCERT study. The *CONgenital Cmv: Efficacy of antiviral treatment in a Randomized controlled Trial (*CONCERT) study aimed to evaluate the efficacy of antiviral therapy in congenital cytomegalovirus (CMV) infection in a randomized clinical trial (clinical trials.gov NCT01655212). The inclusion period was terminated prematurely due to lack of inclusions since parents did not want to randomize for placebo treatment (personal communication). ‘Placebo’ versus ‘treatment’ is different from 3 different treatment strategies. Therefore every particular study design may need a different approach in case of lacking equipoise on the level of the informed patient group. Preferably the informed patient group is involved early in the study design to prevent lack of inclusion in studies. As an example, the future research agenda in JIA will be created according to the James Lind Alliance method [37], to create research that really matters to parents/patients and caregivers and this will potentially increase enrolment in future studies.

## Conclusion

Parental and patient equipoise is important to investigate to enhance recruitment for and retention in studies involving children. In our study, lack of equipoise during enrolment did not reduce study recruitment, due to the fact that preferred treatment was only available within the study. Still, when developing a trial it is important to evaluate whether the research question the physicians want to answer is in line with the preferences of the patient group. By exploring ‘informed patient-group’ equipoise successful recruitment may be enhanced and bias may be avoided.

Lack of equipoise during participation in our long term follow-up trial did not reduce retention in those who were assigned to a less favoured option, probably related to the dynamic treatment-to-target study design. We observed a change for preference towards treatment actually received, possibly explained by favourable outcomes in all three arms [38].

## Additional files

Additional file 1: The three treatment strategies in the BeSt for Kids study. Table representing the initial treatment and treatment steps in the three arms of the BeSt for Kids study. (DOCX 30 kb)

Additional file 2: Information from Parental Informed consent File. This file contains information from the Parental Informed consent File (translated from Dutch). (DOCX 15 kb)

Additional file 3: Questionnaire during enrolment in the BeSt for Kids study (Translated from Dutch). Questionnaire presented to all participants of the BeSt for Kids study during enrolment to evaluate the satisfaction of patient and/or his/her parents with the initial treatment in the study (Phase 1). (DOCX 14 kb)

Additional file 4: Questionnaire Informed Consent Evaluation BeSt for Kids (Phase 2). This questionnaire was used during the interview study (phase 2). (DOC 33 kb)

Additional file 5: **Table S2.** Parental preferences at inclusion (phase 1) and during interview (phase 2), in relation to actual enrolled treatment strategy and **Table S3.** Summary of parental preferences at inclusion (phase 1) and during interview (phase 2) in relation to actual enrolled treatment strategy. **Table S3.** represents, from all interviewed parents, the parental preferences at inclusion (phase 1) and during interview (phase 2), in relation to the actual enrolled treatment strategy. **Table S3.** summarizes the results from **Table S3**. (DOC 61 kb)

## Abbreviations

CMV: Cytomegalovirus
CONCERT study: CONgenital Cmv: Efficacy of antiviral treatment in a randomized controlled trial
DMARD: Disease modyfing antirheumatic drug(s)
JIA: Juvenile idiopathic arthritis
NTR: Dutch trial register
PIF: Patient-subjects information form
RCT: Randomized controlled trial
RF: Rheumatoid factor
