# Supplementary material for: Participation in a single-blinded pediatric therapeutic strategy study for juvenile idiopathic arthritis: are parents and patient-participants in equipoise?
Source: BMC Med Ethics. 2018 Dec 20;19:96. doi: 10.1186/s12910-018-0336-8 (PMC6302476; doi:10.1186/s12910-018-0336-8)
Supplement: Supplementary file 1 — The three treatment strategies in the BeSt for Kids study. Table representing the initial treatment and treatment steps in the three arms of the BeSt for Kids study. (DOCX 30 kb) [file 12910_2018_336_MOESM1_ESM.docx]

Additional file 1 The three treatment strategies in the BeSt for Kids study

| **arm 1**  **Sequential Monotherapy** | **arm 2**  **Combination MTX + 6 wks prednisone** | **arm 3**  **Combination**  **etanercept and**  **MTX** |
| --- | --- | --- |
| MTX 10 mg/m^2^/wk po/sc  Alternative: SSZ 50 mg/kg/day po | MTX 10 mg/m^2^/wk po/sc +  pred 0.5 mg/kg/day tapered to 0 | MTX 10 mg/m^2^/wk po/sc +  ETN 0.8 mg/kg/wk |
|  |  |  |
| MTX 10 mg/m^2^/wk po/sc | MTX 15 mg/m^2^/wk po/sc | MTX 10 mg/m^2^/wk +  ETN 1.6 mg/kg/wk |
|  |  |  |
| MTX 15 mg/m^2^/wk po/sc | MTX 10 mg/m^2^/wk po/sc +  ETN 0.8 mg/kg/wk | Treatment left to treating physician |
|  |  |  |
| MTX 10 mg/m^2^/wk po/sc +  ETN 0.8 mg/kg/wk (max. 50 mg) | MTX 10 mg/m^2^/wk +  ETN 1.6 mg/kg/wk |  |
|  |  |  |
| MTX 10 mg/m^2^/wk +  ETN 1.6 mg/kg/wk (max. 50 mg) | Treatment left to treating physician |  |
|  |  |  |
| Treatment left to treating physician |  |  |

MTX=methotrexate, SSZ=Sulphasalazine, ETN=etanercept, Po=orally, Sc=subcutaneous
