# Supplementary material for: Participation in a single-blinded pediatric therapeutic strategy study for juvenile idiopathic arthritis: are parents and patient-participants in equipoise?
Source: BMC Med Ethics. 2018 Dec 20;19:96. doi: 10.1186/s12910-018-0336-8 (PMC6302476; doi:10.1186/s12910-018-0336-8)
Supplement: Supplementary file 2 — Information from Parental Informed consent File. This file contains information from the Parental Informed consent File (translated from Dutch). (DOCX 15 kb) [file 12910_2018_336_MOESM2_ESM.docx]

Additional file 2

25-08-2017 Questionnaire during enrolment BeSt for Kids study (Translated from Dutch)

This questionnaire is concerning satisfaction of patient and/or his/her parents with the treatment in the study.

Your child/you are participating in the BeSt for Kids study, a study in search for the optimal treatment strategy in patients diagnosed with juvenile idiopathic arthritis.

What we do not know yet, is, if you as patient and parents have a preference for one of the different strategies in the BeSt for Kids study. Because your preference is also very important, we would like to investigate this. Your opinion could be of significant influence on future treatment of patients with juvenile idiopathic arthritis.

Background information:

Patients recently diagnosed with juvenile idiopathic arthritis are treated with anti-rheumatic drugs.

Frequently treatment is started with one medicine, sulfasalazine or methotrexate. If this medicine is not effective enough, treatment is changed to the next anti-rheumatic drug. However, some indications show that direct treatment with a combination of anti-rheumatic drugs could be more successful. Besides that, since some time a new medicine is available administered by injection, etanercept (Enbrel), which is clearly beneficial in patients with chronic, severe juvenile arthritis not responding to traditional treatment.

What was the treatment group that you **do** hoped to be allotted to?

- No
- Yes we hoped to be allotted to arm 1
- Yes we hoped to be allotted to arm 2
- Yes we hoped to be allotted to arm 3

What was the treatment group that you hoped **not** to be allotted to?

- No
- Yes we hoped not te be allotted to arm 1
- Yes we hoped not to be allotted to arm 2
- Yes we hoped not to be allotted to arm 3
