# Supplementary material for: Participation in a single-blinded pediatric therapeutic strategy study for juvenile idiopathic arthritis: are parents and patient-participants in equipoise?
Source: BMC Med Ethics. 2018 Dec 20;19:96. doi: 10.1186/s12910-018-0336-8 (PMC6302476; doi:10.1186/s12910-018-0336-8)
Supplement: Supplementary file 3 — Questionnaire during enrolment in the BeSt for Kids study (Translated from Dutch). Questionnaire presented to all participants of the BeSt for Kids study during enrolment to evaluate the satisfaction of patient and/or his/her parents with the initial treatment in the study (Phase 1). (DOCX 14 kb) [file 12910_2018_336_MOESM3_ESM.docx]

Additional file3

Relevant information from parental informed consent brochure

Why this study

The goal of this study is to determine which of three treatment strategies is the best for a child with oligo or polyarticular juvenile idiopathic arthritis.

It is not the point which individual medication is superior but we want to compare if it is better to give the medication one after another or to start with a combination or directly with maximum treatment. This comparison is relevant to study if early aggressive treatment is better able to prevent joint damage in the future. At the moment we cannot predict the course of the disease in an individual patient.

We know that if a child has only a few or even one joint with arthritis (oligoarticular course) at the start of disease, problems later in life can be comparable with a child with many joints with arthritis at the start (polyarticular course). Maybe it is superior to treat the disease from the start maximally and then, if all arthritis is over, taper medication.

All medication used in this study is already in use in the treatment of children with juvenile arthritis.

What does the study entail?

If you decide to participate in the study your child will be randomized in one of the following 3 options:

Option 1: treatment with antirheumatic drugs sequentially until arthritis is over. The first medicine is named sulfasalazine or methotrexate (tablets, first low dose, later high dose if necessary) and the third medicine is etanercept (administered through an injection, first normal dose, possible higher dose later)

Option 2: the antirheumatic drug methotrexate is combined with prednisolone and if this is not sufficient or in case of (severe) side effects treatment is changed to etanercept.

Option 3: etanercept is started directly combined with methotrexate.

Risks and concerns

There are pros and cons to each of the three treatments strategies mentioned. In short, the most experience exists with the first and second option. The medication has been known for a long time, so the short and long term effects and adverse effects are known. From the third treatment strategy we know that it can quickly have a good effect on children with juvenile arthritis, especially when many joints are involved in inflammation. The side effects of this treatment on the long-term are not well known yet, but by stopping the medication soon after joint inflammation disappears, the child is exposed relatively short.
