# Supplementary material for: Participation in a single-blinded pediatric therapeutic strategy study for juvenile idiopathic arthritis: are parents and patient-participants in equipoise?
Source: BMC Med Ethics. 2018 Dec 20;19:96. doi: 10.1186/s12910-018-0336-8 (PMC6302476; doi:10.1186/s12910-018-0336-8)
Supplement: Supplementary file 4 — Questionnaire Informed Consent Evaluation BeSt for Kids (Phase 2). This questionnaire was used during the interview study (phase 2). (DOC 33 kb) [file 12910_2018_336_MOESM4_ESM.doc]

Additional file 4

**Questionnaire Informed Consent Evaluation BeSt for Kids**

**1.General questions and demographic information**

1. Sex patient

□ Male □ Female

2. Date of birth patient:

3. Date of birth parents:

Father:

Mother:

4. Place of residency of patient and family

5. Composition of the family

□ Only child

□ Brothers and sisters, that is ......

□ Parents together

□ Parents divorced

6. Working Activities of parents:

Father:

Mother:

7. Highest education level father :

Primary school / lower level high school

Middle level high school

Advanced vocational / university

8. Highest education level mother:

Primary school / lower level high school

Middle level high school

Advanced vocational / university

9. Nationality parents:

□ Dutch

□ Other, that is,.......

10. Religion of parents

□ Christianity

□ Jewish

□ Islam

□ No religion

□ Other, that is....

**2. Retainment of information concerning the trial**

1.What trial is your child participating in?

a. BeSt for Kids

b. Reumatism for Kids

c. JIA for Kids

d. I don’t know

2. What is the goal of the trial?

a. To test new medication for children with certain types of Juvenile Idiopathic Arthritis

b. To investigate the best treatment strategy from three possibilities for children with certain types of juvenile idiopathic arthritis

c. To research what particular drug is the best treatment for children with JIA

d. I don’t know

3. How many strategies exist in the trial

a. 5

b. 3

c. 4

d. I don’t know

4. Who decided which strategy your child received?

a. The treating physician

b. This was assigned by lot

c. The principal investigator

d. I don’t know

5. Have you been informed about risks concerning trial participation, and if yes what risks?

6. Have you been informed about benefits of trial participation, and if yes what benefits?

7. Are there any extra procedures (like blooddraws, visits to the outpatient clinic) that your child would not receive if not participating in the trial?

8. What is the duration of the trial?

a. 1 year

b. 2 years

c. 3 years

d. I don’t know

9. Are data of your child preserved?

a. Yes

b. Yes, as long as permission was given

c. No

d. I don’t know

10. Is the treating physician aware of the treatment strategy of your child?

a. No this is a blinded trial

b. Yes the physician is informed

c. I don’t know

11. Are you allowed to withdraw at all times from the trial?

a. Yes

b. No

c. I don’t know

12. Does this have consequences for the treatment of your child?

a. Yes

b. No

c. I don’t know

13. Is it possible to discuss issues with an independent physician?

a. Yes

b. No

c. I don’t know

14. If yes, do you know who it is?

a. No I don’t know

b. Yes, that is…..

15. Did you understand the provided information at that time?

a. Yes it was clear

b. Yes after extra verbal explanation

c. No it was not clear

d. I don’t know

16. Have you been asked if everything was clear to you?

a. Yes

b. No

c. I don’t know

17. Did you have enough time to answer questions?

a. Yes

b. No

18. Did you receive information to take home?

a. Yes

b. No

c. I don’t know

19. Was this information sufficient?

a. Yes

b. Too much information

c. Too little information

20. Did you experience enough time to think about participation in the trial?

a. Yes, enough

b. Yes but could have been more

c. No, not enough

21. Who finally decided to participate in the trial?

a. the physician

b. the physician and parents

c. the physician, parents and patient

d. the parents

e. the parents and patient

**3. The next questions are to increase our perception of your considerations regarding the treatment**

1. What was your preferred strategy at the moment of trial inclusion?

In other words: did you wish your child to be assigned to a particular treatment strategy?

2. Can you explain why?

3. What is your preferred strategy now?

4. Can you explain why?

5. Are you satisfied with the given treatment?

6. To your opinion, did the physician have a preferred strategy?

**4. Suggestions for improvement and questions**

1. What was your impression of the conversation about trial participation of your child?

2. Are you satisfied with the communication with the physician during the process towards participation in the trial?

3. What was your main reason for participation in the trial?

4. Would you like to stay informed about the results of the trial?

5. Do you have suggestions for improvement of the informed consent procedure?

6. Do you have any more questions or remarks?
