# Supplementary material for: Participation in a single-blinded pediatric therapeutic strategy study for juvenile idiopathic arthritis: are parents and patient-participants in equipoise?
Source: BMC Med Ethics. 2018 Dec 20;19:96. doi: 10.1186/s12910-018-0336-8 (PMC6302476; doi:10.1186/s12910-018-0336-8)
Supplement: Supplementary file 5 — Table S2. Parental preferences at inclusion (phase 1) and during interview (phase 2), in relation to actual enrolled treatment strategy and Table S3. Summary of parental preferences at inclusion (phase 1) and during interview (phase 2) in relation to actual enrolled treatment strategy. Table S3. represents, from all interviewed parents, the parental preferences at inclusion (phase 1) and during interview (phase 2), in relation to the actual enrolled treatment strategy. Table S3. summarizes the results from Table S3. (DOC 61 kb) [file 12910_2018_336_MOESM5_ESM.doc]

Additional file 5

| Parent number | Preferred strategy at inclusion  (phase 1) | Actual Treatment strategy | Preferred Strategy during interview  (phase 2) | Change of preference  To actual treatment strategy | Change of preference  to  Arm 3 |
| --- | --- | --- | --- | --- | --- |
| 1 | 2 | 1 | 1 | X |  |
| 2 | No preference | 2 | 3 |  | X |
| 3 | 3 | 3 | 3 |  |  |
| 4 | No preference | 2 | 2 | X |  |
| 5 | 2 | 2 | 2 |  |  |
| 6 | No preference | 3 | 3 | X | X |
| 7 | 3 | 1 | 3 |  |  |
| 8 | 3 | 1 | 3 |  |  |
| 9 | 1 | 1 | 3 |  | X |
| 10 | No preference | 3 | 1 |  |  |
| 11 | 3 | 1 | 3 |  |  |
| 12 | 3 | 3 | 3 |  |  |
| 13 | 3 | 1 | 1 | X |  |
| 14 | 3 | 1 | 3 |  |  |
| 15 | 3 | 1 | 1 | X |  |
| 16 | No preference | 3 | 3 | x | X |
| 17 | No preference | 1 | 1 | X |  |
| 18 | 3 | 3 | 3 |  |  |
| 19 | No preference | 2 | 1 |  |  |
| 20 | 3 | 3 | 3 |  |  |
| 21 | No preference | 3 | 1 |  |  |
| 22 | 3 | 2 | 3 |  |  |
| 23 | 3 | 1 | 3 |  |  |

Table S2 Parent preferences at inclusion (phase 1) and during interview (phase 2), in relation to actual enrolled treatment strategy.

|  | No preference | Arm 1 | Arm 2 | Arm 3 |
| --- | --- | --- | --- | --- |
| Initial preference  (phase 1) | 8 | 1 | 2 | 12 |
| Actual treatment strategy | NA | 10 | 5 | 8 |
| Preference during interview  (phase 2)  Compatible with actual treatment strategy | 0 | 7  4 | 2  2 | 14  6 |

Table S3 Summary of initial preferences (phase 1), actual enrolled arm and preference during the interview (phase 2). NA=not applicable.
